# A case report of retiform hemangioendothelioma as pleural nodules with literature review

**DOI:** 10.1186/s13000-015-0433-7

**Published:** 2015-10-26

**Authors:** Qingqing Liu, Ruoyun Ouyang, Ping Chen, Rui Zhou

**Affiliations:** Department of Internal Medicine, Second Xiangya Hospital of Central South University, 139 Renming Rd, Changsha, Hunan 410011 PR China

**Keywords:** Retiform hemangioendothelioma, Pleural effusion, Breast cancer, CD31, CD34, D2-40

## Abstract

Retiform hemangioendothelioma (RH) is a rare low-grade variant of angiosarcoma mostly reported on dermis or subcutaneously. A 30-year-old woman suffering from dry cough, dyspnea and pleural effusion has been described. Distinctive symptoms and lesions on high resolution computed tomography (HRCT) scan and common histological, immunological feature are discussed. Diagnosis was made by thoracoscopy as RH.

## Background

RH is known as a rare kind of low-grade malignant angiosarcoma with high rate of local recurrence and low potential of metastasis which is a sparsely distributed disease counting 35 cases (Table 1) whereas none of them is related to pleura or lung [1]. Given the lacking information of pleural RH, its clinical manifestation, HRCT presentation and thoracoscopic appearance of a female patient are presented. The definitive diagnosis was made due to pathological observation and immunochemical staining. The prognosis is unpredictable since the tumor can affect multiple organs [2]. Unfortunately, consensus has not been reached on if there is effective treatment to this tumor.

**Table 1:** Summary of clinical features of reported RH cases

Author (N)	Age/Gender	Site	Immunochemistry	Metastasis	Treatment	Survival
Calonje (15)	9–78/9 F,6 M	6:lower limbs	ND	1/15	ND	ND
		4:upper limbs				
		3:Trunk				
		1:Penis				
		1:Scalp				
Fukunaga (1)	75/F	Lower limb	CD31, vimentin, UEA-1 (all), CD34, f-VIII (part)	0/1	ND	ND
Duke (1)	30/F	Upper limb, trunk	CD31, f-VIII	0/1	Excision	>10 years
Samz-Trelles (1)	11/M	Lower limb	Vimentin, f-VIII	0/1	Excision	>4 years
Schommer (1)	73/F	Trunk	CD31,UEA-1, f-VIII	0/1	Excision, RT, IT	>1 year
Darouti (1)	32/F	Lower limb	f-VIII	ND	ND	>1.5 years
Tan (1)	19/F	Lower limb	ND	0/1	Excision	>14 months
Ioannidon (1)	55/F	Upper limb	CD31	0/1	Excision	>4 years
Parson (1)	17–71/4 F,0 M	1:upper limb	CD31(4/4),	ND	ND	ND
		1:trunk	D2-40(1/4),			
		1:head	VEGFR3(0/4)			
		1:lower limb				
Bhutoria (1)	35/F	Trunk	ND	Lymph node	Excision	ND
Emberger (1)	17/M	Trunk	CD31,D2-40	0/1	Excision	>3 years
Zhang (1)	61/F	Head	CD34, f-VIII, vimentin	0/1	Excision(twice)	6 months
Aydıngöz (1)	60/F	Lower limb	ND	0/1	Excision	>2 years
Hirsh (1)	44/M	Trunk	CD31	0/1	Resection,chemoradiation	>36 months
Keiler (1)	11/F	Upper limb	ND	0/1	Excision	ND
O’Duffy (1)	18/M	Head	ND	Lymph node	Excision, RT	ND
Albertini(1)	6/F	Trunk	D2-40	Lung	Excision	6 months
Choi (1)	20/M	Upper limb	f-VIII	ND	Excision	ND
Couceiro (1)	50/F	Upper limb	ND	ND	Excision	>4 years
Mota (1)	26/F	Trunk	CD31, D2-40	ND	Excision	ND
Al-Faky	9/F	Head	CD31, CD34, D2-40, f-VIII	ND	Excision	>6 years

*ND* not documented, *M* male, *F* female, *UEA-1* ulex europaeus agglutinin 1, *f-VIII* factor VIII-related antigen, *RT* radiotherapy, *IT* immunotherapy

## Case presentation

### Clinical history and radiology

**Fig. 1:**
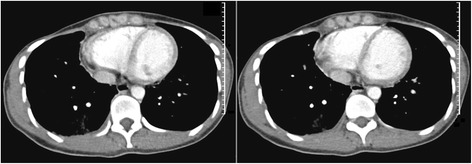
HRCT scan of the patient before thoracoscopy. Small amount of unilateral pleural effusion was detected on the right side of the patient. None visible pleural nodes were reported. (Opacity showed in the patient’s right lung which has been proved to be bacterial infection because it disappeared after treatment of antibiotics)

A 30-year-old female patient was admitted to pulmonary department because of dyspnea and dry cough denying chest pain, hemoptysis, fever, weight loss or other systemic symptoms. Her symptoms were stable and had not been given any medication prior to admission. Her chest X-ray by local hospital showed pleural effusion on the right side. She received modified radical mastectomy in 2006 removing a breast mass on her right chest. The biopsy confirmed a diagnosis of poorly-differentiated invasive ductal carcinoma with axillary lymph nodes metastasis therefore the patient received adjuvant chemotherapy and radiotherapy. Her father died of liver cancer. The rest of her personal history, social history and the review of systems were unremarkable. Decreased vocal fremitus and dullness of percussion on her right thorax were detected in physical examination. No enlarged superficial lymph nodes were palpated and laboratory tests revealed normal level of carcino-embryonic antigen (CEA). Her ultrasonic examination of abdomen and pelvis cavity alarmed no metastatic signs. The patient’s HRCT results showed merely pleural effusion on the right side (Fig. 1). No residual mass, enlarged lymph nodes of hilar or lesion on pleura were observed. Laboratory tests of her pleural effusion samples contained erythrocytes and leukocytes and it was exudates. Furthermore, cytological examination reported large amount of lymphocytes without atypical or carcinomatous cells. Scattered greyish variously sized nodes were discovered on costal pleura in thoracoscopy. The lesion presented themselves as exophytic masses surrounded by indistinctive borders with a tendency to fuse together. Pulmonary and diaphragmatic pleura were free from infiltration. Rechecking of chest X-ray complied with her former HRCT scan.

### Histology and immunohistochemistry

**Fig. 2:**
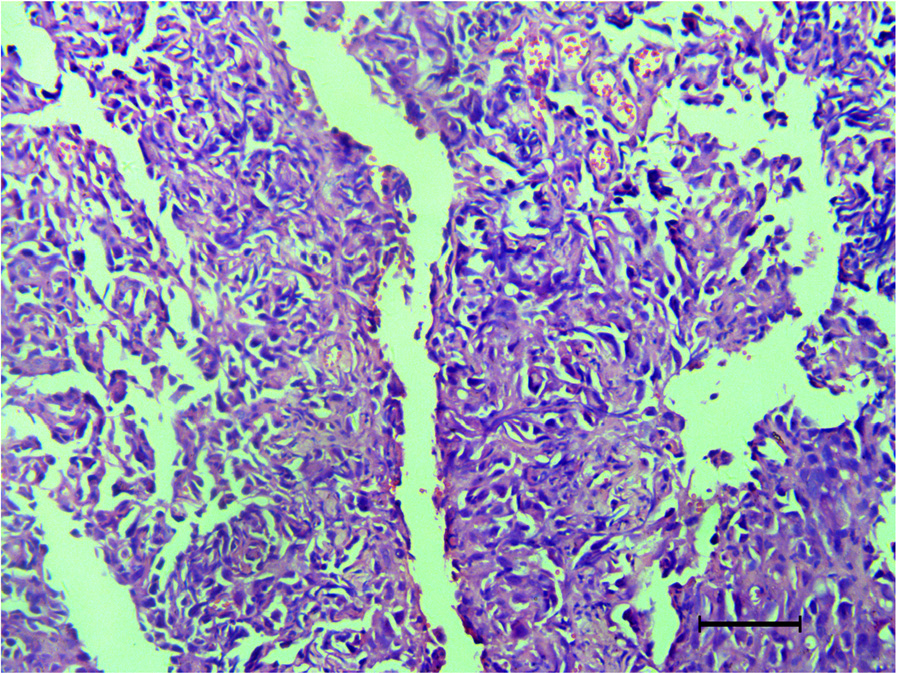
Hematoxylin and eosin staining of the patient’s sample. Hematoxylin and eosin staining presented atypical cells forming disarranged and abnormal lumina. Some of the “vessels” were complete and contained erythrocytes and main part of the tumor consisted of zigzag slits. No atypia or mitosis of nuclei showed in the cells meanwhile cytoplasm was insufficient

**Fig. 3:**
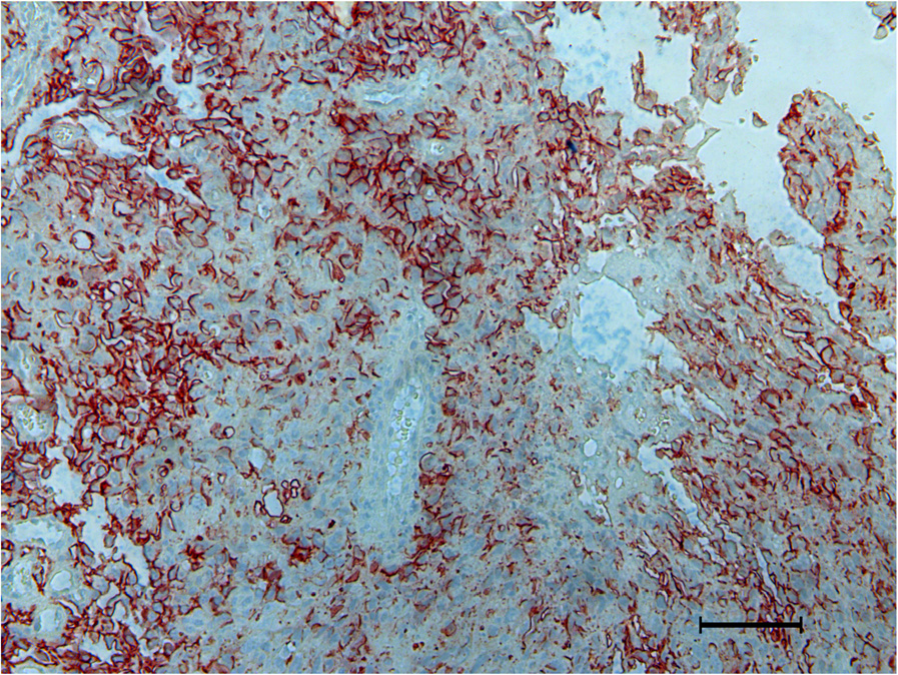
Immunohistochemical staining of the patient’s sample. Immunohistochemical staining for D2-40 expression was positive

Histological and immunological examination of biopsy specimen pictured disarranged, jagged, blood vessels of thin walls and slit-like fissures were prevailing. At higher magnification the vessels’ walls were lined with highly nuclei/cytoplasm ratioed endothelial cells with minimal cytological atypia and mitosis. Atypical cells protruding into lumina mimicking “hobnail” appearance or endothelial papillae was absent as well as their invasion into adjacent tissues. Infiltration of lymphocytes was also rare (Fig. 2). Combined with immunohistochemical staining positive for CD31, CD34, D2-40 (Fig. 3) and negative for CrebB-2, GCDFP-15, CEA, CA15-3, S-100, Ki-67 and myogenin, which confirmed the diagnosis and ruled out the possibility of breast cancer and other diagnosis, a diagnosis of RH was established.

### Results

According to our knowledge and clinical experience, endostatin was recommended for treatment. The patient revisited 6 months later with relieved symptoms when her HRCT showed diminished and encapsulated pleural effusion after receiving symptomatic treatment and passed away at home in 2013.

## Discussion

Hemangioendothelioma (HE), an intermediate lesion between hemangioma and angiosarcoma, has been incipiently delineated as a kind of vascular tumor highlighted by its marginal biological features [3]. It generally appears on skin or in soft tissues and extensively includes HE of epithelioid, kaposiform, retiform, composite and pseudomyogenic (also known as epithelioid sarcoma-like HE) subgroups and papillary intralymphatic angioendothelioma (also known as Dabska tumor). Malignancy of pseudomyogenic HE has recently been re-recognized [4]. Definitive diagnosis of varied types of this tumor is based on histopathlological morphology and immunohistochemical markers [5]. HE on pleura are limitedly documented as majority of them have been confirmed as epithelioid hemangioendothelioma (EH). Though EH favorably links to lung and pleura according to published cases, there has never been a description of its counterpart-retiform hemangioendothelioma on pleura.

Retiform hemangioendothelioma is a low-grade variant angiosarcoma which has been first introduced by Calonje in 1994 [6]. Hyperchromatic nuclei without mitosis suggests its intermediate characters [7] and indicates discrepant prognosis.

RH demonstrates a predilection for female adults [6]. The majority of RH cases have been described as lesions on trunk or limbs [8] whereas penis and scalp were occasionally involved [9]. Features of RH’s lesion vary among individual patients expressing as masses, which is hyperhidrotic, or erosion [8, 10, 11]. Exophytic pattern seems to be more prevailing [8]. All the lesions in this patient were surrounded by “poor circumscription” as a presentation of its histological character.

Histological diagnosis lays the foundation of clinical diagnosis also plays the key role in differentiating RH from angiosarcoma and other subtypes of hemangioendothelioma. RH comprises of blood vessels ”weaving a net” by infiltrating into bundles of collagen. Endothelial cells fashion intraluminal papillea with scarce cytoplasm [8, 12]. However, pathological sample in this case for histological examination may accidentally be a single component of “composite hemangioendothelioma” which contains retiform HE, epithelioid HE, angiosarcoma, spindle cell hemangioma, lymphangioma, arteriovenous malformation [13] or part of these. This assumption is drawn from the fact that excision of gross sample has not be performed taking the fact that EH tends to form in lung into consideration [14].

Histochemical markers of RH include CD31, D2-40, CD34, factor VIII-related antigen [15, 16] among which CD34, factor VIII-related antigen have been consistently reported. CD34 and CD31 symbolize endothelial lineage and D2-40 expresses on other kinds of lymphatic hemangioendothelioma or hemangioma [17, 18]. Controversy, nevertheless, has been aroused by Amy Parson's demonstration of 3 negative cases of D2-40 in 4 RH patients [19].

Though histological and clinical features permits differential diagnosis between RH and other vascular tumors [20], it shares a number of similarities with hobnail hemangioma which has been considered as it benign counterpart. However, hobnail hemangioma features shortage of papillae protruding into lumina and restricted location [21] while prominent lymphatic infiltration and extensive lesion (dermis and subcutis) are widely applied to make the RH diagnosis [22]. Moreover, definitive diagnosis of a papillary lymphatic angioendothelioma with partial “netlike” camouflage and D2-40 positivity was in arguement [21].

The etiology of RH remains obscure. Latent relations between malignant medical history and RH should be elucidated [2, 9]. In this case no evidence supports the internal link between RH occurence and the past radiotherapy.

No tumor-related [8] deaths in RHs are observed under the circumstance of low metastasis. However, a locally invasive RH in China and a metastatic RH in a six-year-old girl turned out to be lethal [23]. Although adjuvant immunotherapy and radiotherapy  have been proved to be effective in several cases, none agreed tumor-responding treatments are recommended unless a complete surgical excision is performed [24–27]. Amanda and her colleagues, however, proposed specific appearance on dermoscopy as practical assistance to diagnosis and therapy [28].

## Conclusions

In summary, a case of RH situating on pleura has been depicted which exhibited peculiar pathological, immunological features and lesions. It has been reported as intrathoracic nodules predisposing a patient to unilateral pleural effusion. However discussion has been placed on the causes and treatment of RH. It implies that RH on location other than skin or subcutis may lead to corresponding symptoms or even threatened survival.

## Consent

Written informed consent was obtained from the patient for publication of this Case Report and any accompanying images. A copy of the written consent is available for review by the Editor-in-Chief of this journal.

## Abbreviations

RH: Retiform hemangioendothelioma
CEA: Carcino-embryonic antigen
HRCT: High resolution computed tomography
HE: Hemangioendothelioma
EH: Epithelioid hemangioendothelioma
